# Hepatitis B Vaccine Responsiveness and Clinical Outcomes in HIV Controllers

**DOI:** 10.1371/journal.pone.0105591

**Published:** 2014-08-21

**Authors:** Jason F. Okulicz, Octavio Mesner, Anuradha Ganesan, Thomas A. O’Bryan, Robert G. Deiss, Brian K. Agan

**Affiliations:** 1 Infectious Disease Clinical Research Program, Uniformed Services University of the Health Sciences, Bethesda, Maryland, United States of America; 2 Infectious Disease Service, San Antonio Military Medical Center, San Antonio, Texas, United States of America; 3 Infectious Disease Service, Walter Reed National Military Medical Center, Bethesda, Maryland, United States of America; 4 Infectious Disease Clinic, Naval Medical Center San Diego, San Diego, California, United States of America; Centers for Disease Control and Prevention, United States of America

## Abstract

**Background:**

Hepatitis B virus (HBV) vaccine responsiveness is associated with reduced risk of AIDS or death in HIV-infected individuals. Although HIV controllers (HIC) typically have favorable immunologic and clinical characteristics compared to non-controllers, vaccine responsiveness has not been studied.

**Methods and Findings:**

In the U.S. Military HIV Natural History Study, HBV vaccine response was defined as antibody to hepatitis B surface antigen (anti-HBs) ≥10 IU/L after last vaccination. For determination of vaccine responsiveness, HIC (n = 44) and treatment-naïve non-controllers (n = 476) were not on highly active antiretroviral therapy (HAART) when vaccinated while treated non-controllers (n = 284) received all HBV vaccine doses during viral load (VL)-suppressive HAART. Progression to AIDS or death was also compared for all HIC (n = 143) and non-controllers (n = 1566) with documented anti-HBs regardless of the timing of HBV vaccination. Positive vaccine responses were more common in HIC (65.9%) compared to HAART-naïve non-controllers (36.6%; P<0.001), but similar to non-controllers on HAART (59.9%; P = 0.549). Factors associated with vaccine response for HIC compared to HAART-naïve non-controllers include HIC status (OR 2.65, 95% CI 1.23–5.89; P = 0.014), CD4 count at last vaccination (OR 1.28, 1.15–1.45 for every 100 cells/uL; P<0.001), and number of vaccine doses administered (OR 0.56, 0.35–0.88; P = 0.011). When HIC were compared to non-controllers on HAART, only CD4 count at last vaccination was significant (OR 1.23, 1.1–1.38 for every 100 cells/uL; P<0.001). The rate of AIDS or death per 100 person/years for HIC compared to non-controllers was 0.14 (95% CI 0–0.76) versus 0.98 (95% CI 0.74–1.28) for vaccine responders and 0 (95% CI 0–2.22) versus 4.11 (95% CI 3.38–4.96) for non-responders, respectively.

**Conclusions:**

HIC have improved HBV vaccine responsiveness compared to treatment-naïve non-controllers, but similar to those on VL-suppressive HAART. Progression to AIDS or death can be predicted by HBV vaccine responder status for non-controllers, however these events are rarely observed in HIC.

## Introduction

Elite and viremic controllers, collectively termed HIV controllers (HIC), are an uncommon subgroup of HIV-infected individuals with the ability to naturally suppress plasma viral load (VL) in the absence of highly active antiretroviral therapy (HAART). Elite controllers typically suppress VL below the limit of detection of clinical assays while viremic controllers exhibit a lesser degree of virologic control with low level viremia. Elite and viremic controllers comprise <1% and approximately 3% of persons in most HIV cohorts, respectively [1]–[3]. Although defined by virologic criteria, HIC status is typically associated with improved clinical outcomes comparable to individuals on VL-suppressive HAART, including higher CD4 counts and reduced risk of developing AIDS and death [1], [4]–[6].

To determine the mechanisms responsible for spontaneous virologic control, HIC are intensely studied with the anticipation of developing novel treatment strategies and possibly a therapeutic vaccine for the treatment of HIV. One aspect of HIC that has not been sufficiently studied is immune response to vaccinations. Since HIC status is associated with more favorable functional immunity, enhanced responses to vaccinations may be expected. The hepatitis B virus (HBV) vaccine has several advantages for studying vaccine response in HIV-infected persons. In contrast to some other vaccines, such as the pneumococcal polysaccharide vaccine which is a T-cell independent antigen, HBV vaccine may provide a more complete assessment of B-cell and T-cell function. A positive response to HBV vaccination requires T-cell processing, but also other aspects of immune function including antigen presentation of the peptide-based vaccine and B-cell activity [7]–[10]. HBV vaccination is also recommended for all HIV-infected individuals without prior immunity and serologic response can be routinely assessed by antibody detection (anti-HBs)[11]. Finally, HBV vaccine has prognostic value in the setting of HIV infection as vaccine responders have been shown to have a reduced risk of developing AIDS and death, including those with CD4 counts >500 cells/uL [12].

HIV-infected patients have diminished responsiveness to HBV vaccination, ranging from 20–62% compared to 90% in HIV-uninfected persons [13]–[15]. Despite the diminished response rates, there are several HIV disease-related factors associated with improved HBV vaccine responses, including CD4 cell count >350 cells/uL and use of effective HAART resulting in VL suppression and subsequent immune reconstitution [13], [16], [17]. HIC typically possess many of these factors in the absence of HAART, however HBV vaccine response in the setting of spontaneous virologic suppression has not been studied. We investigated HBV vaccine responses in HIC compared to non-controllers with or without VL-suppressive HAART in the US Military HIV Natural History Study (NHS). Since non-response to HBV vaccine has been associated with HIV disease progression in those with relatively preserved CD4 counts, we also studied the longitudinal development of AIDS or death outcomes for HIC and non-controllers with documented HBV vaccine responses.

## Materials and Methods

The NHS is a prospective observational cohort of over 5500 active duty military members, dependents, and beneficiaries with HIV infection followed in the military healthcare system since 1986 [18]. Clinical evaluations occur approximately every 6 months at selected military treatment facilities in the United States, with systematic data collection including demographic characteristics, laboratory and treatment records, and reports of clinical events with medical record confirmation. All NHS participants provided written informed consent and were ≥18 years of age. This study was approved by the Uniformed Services University of the Health Sciences Infectious Disease Institutional Review Board. The NHS provides open access to data by email request through its concept review process (IDCRP@IDCRP.org).

### Comparison Groups

HIC are composed of elite and viremic controllers as previously described [1], [19]. Elite controllers were defined as having 3 or more VL tests below the limit of detection of the assay over a period of ≥12 months in the absence of HAART. Viremic controllers were characterized by a lesser degree of virologic control, defined as ≥3 VLs ≤2000 copies/mL over a period of ≥12 months in the absence of HAART. Non-controllers were cohort participants not meeting HIC definitions.

### HBV Vaccine Seroresponses

We selected 2 distinct groups of non-controllers for HBV vaccine response analyses. Since HBV vaccine responses are greatly diminished when vaccination occurs after HIV infection, we first compared HIC and HAART-naïve non-controllers receiving all vaccine doses after HIV diagnosis. Both groups were required to remain HAART-naïve through the date of last HBV vaccination and evaluation of vaccine response. Since HIC have similar characteristics to HIV-infected individuals on VL-suppressive HAART, a second group of non-controllers who received all HBV vaccinations after HAART initiation was also studied. Non-controllers on VL-suppressive HAART were defined as achieving VL<400 copies/mL within the first 6 months of their initial HAART regimen with continued VL suppression during follow-up for determination of vaccine responsiveness. Participants with clinical or serologic evidence of prior HBV infection were excluded. Since HBV vaccine responsiveness is significantly greater in those without HIV infection, participants with receipt of any HBV vaccine doses prior to HIV diagnosis were excluded for the analysis of HBV vaccine response. A positive HBV vaccine response was defined as anti-HBs ≥10 IU/L measured ≥1 month after the last vaccine dose administered, while non-response was defined by anti-HBs <10 IU/L. To determine whether positive HBV vaccine responses occur more rapidly in HIC compared to non-controllers, a subgroup analysis was performed for participants with an anti-HBs determination ≥1 month after the first HBV vaccination and prior to the receipt of additional vaccine doses.

### Longitudinal Outcomes of AIDS or Death by HBV Vaccine Response Status

HBV vaccine responsiveness requires several immune pathways and can serve as an indicator of functional immune responses in the host. To expand upon our previous observation that HBV vaccine seroresponse is a predictor of HIV disease progression [12], we studied all HIC and non-controllers with a documented anti-HBs determination for the time to development of new AIDS outcomes or death. For those receiving any or all HBV vaccinations after HIV infection, the date of first anti-HBs ≥1 month after last vaccination was defined as baseline and the value was used to assign responder status. For participants vaccinated prior to HIV infection, the first anti-HBs test after HIV infection was used for response category assignment and baseline was the date of HIV diagnosis. Participants with prior HBV infection were excluded. AIDS outcomes were defined by 1993 Centers for Disease Control and Prevention criteria [20].

### Statistical methods

All baseline comparisons were made using either t-, Wilcoxon, chi-squared, or Fisher’s exact tests. For continuous variables, normality was first assessed using Shapiro-Wilk test. T-tests were used for normal variables and Wilcoxon for non-normal variables. For categorical variables, chi-squared tests were used if cell expected values were 10 or greater; otherwise, Fisher’s exact test was used. For assessing the adjusted effect of covariates on HBV vaccine response, logistic regression was used. Wald tests were used to calculate p-values and confidence intervals. Cox proportional hazards model was used to assess factors associated with time to AIDS or death from last vaccination or assignment of HBV response category where applicable. Included variables were chosen by clinical significance and the model was stratified by era of HIV diagnosis (before or after 1996). Weibull regression was used as a sensitivity analysis. Covariates for logistic regression and Cox proportional hazards model were selected collaboratively using clinically relevant and statistically significant variables. Kaplan-Meier curves with log-rank tests were used to separately compare the effect of HIC status and HBV vaccine responsiveness on time to new AIDS diagnosis or death. Rates and exact Poisson confidence intervals were calculated for each group. All P-values were two-sided with P<0.05 considered statistically significant. Analyses were performed using R statistical programming language (2.13.2).

## Results

### HBV Vaccine Seroresponses

A total of 44 HIC met inclusion criteria for the HBV vaccine response analysis, including 2 elite and 42 viremic controllers, and 476 HAART-naïve non-controllers (Table 1). Both groups were predominantly male with similar demographic characteristics except HIC had a larger proportion of African Americans (45.5%) while the greatest proportion of non-controllers were European American (44.7%). The median log_10_ VL was lower for HIC at both HIV diagnosis (3.2, IQR 2.6–3.8 vs 4.3, IQR 3.8–4.7; P<0.001) and at last HBV vaccination (3.1, IQR 2.6–3.5 vs 4.1, 3.7–4.6; P<0.001) compared to HAART-naïve non-controllers and CD4 counts were higher for HIC at both time points (614, IQR 519–814 cells/uL vs 510, IQR 392–676 cells/uL; P = 0.007 and 617, IQR 520–761 cells/uL vs 452, IQR 348–588 cells/uL; P<0.001, respectively). There were similar proportions of AIDS diagnoses prior to last vaccination for HIC vs HAART-naïve non-controllers (13.6% vs 19.3%; P = 0.470). The time from HIV diagnosis to receipt of last HBV vaccination was similar (approximately 18 months) and most participants received ≥3 doses of HBV vaccine in both groups.

**Table 1:** Baseline Characteristics of HIV Controllers and Non-controllers With or Without Viral Load-Suppressive HAART for HBV Vaccine Seroresponse.

Characteristic	HIV Controllers	HAART-naïve Non-controllers	P-value^a^	Non-controllers on HAART	P-value^b^
Number of Participants (N)	44	476		284	
Age at HIV Diagnosis (Years)	27 (22–35)	28 (24–32)	0.948	30 (25–37)	0.04
Gender			0.216		0.715
Male	35 (79.5%)	416 (87.4%)		236 (83.1%)	
Female	9 (20.5%)	60 (12.6%)		48 (16.9%)	
Race/Ethnicity			0.047		0.068
European American	13 (29.5%)	217 (45.5%)		127 (44.7%)	
African American	20 (45.5%)	194 (40.8%)		118 (41.5%)	
Hispanic/Other	11 (25%)	65 (13.7%)		39 (13.7%)	
CD4 Count at HIV Diagnosis (Cells/uL)	614 (519–814)	510 (392–676)	0.007	363 (230–543)	<0.001
VL at HIV Diagnosis (Log_10_ Copies/mL)	3.2 (2.6–3.8)	4.3 (3.8–4.7)	<0.001	4.8 (4.2–5.1)	<0.001
Age at Last Vaccination (Years)	31 (26–38)	31 (27–36)	0.816	37 (29–45)	<0.001
AIDS Before Last Vaccination	6 (13.6%)	92 (19.3%)	0.47	110 (38.7%)	0.002
Number of Vaccine Doses			0.878		0.521
1 or 2	21 (47.7%)	227 (47.7%)		154 (54.2%)	
≥3	23 (52.3%)	249 (52.3%)		130 (45.8%)	
Time from HIV Diagnosis to Last Vaccination (Months)	17.0 (7.0–48.0)	18.0 (7.6–41.0)	0.778	42 (17.0–107.0)	<0.001
CD4 Count at Last Vaccination (Cells/uL)	617 (520–761)	452 (348–588)	<0.001	524 (380–714)	0.023
VL at Last Vaccination (Log_10_ Copies/mL)	3.1 (2.6–3.5)	4.1 (3.7–4.6)	<0.001	2.0 (1.7–2.6)	<0.001
HCV Diagnosis Before Last Vaccination	1 (2%)	2 (0.4%)	0.233	3 (1.1%)	0.44
Time from Last Vaccination to Anti-HBs Test (Months)	5.6 (3.8–7.1)	5.3 (2.0–8.0)	0.782	5.5 (3.1–8.3)	0.493

HAART, highly active antiretroviral therapy; HBV, hepatitis B virus.

a P-value, HIV controllers vs. HAART-naïve non-controllers.

b P-value HIV controllers vs. non-controllers on HAART.

AIDS, acquired immunodeficiency syndrome; VL, viral load; HCV, hepatitis C virus; anti-HBs, antibody to hepatitis B surface antigen.

Data are number (%) or median (Interquartile Range).

Positive HBV vaccine seroresponse defined as anti-HBs ≥10 IU/L measured ≥1 month after last HBV vaccination.

The group of 284 non-controllers on VL-suppressive HAART had similar demographic characteristics as HIC except for greater median age at last vaccination (37 years, IQR 29–45 vs 31 years, IQR 26–38; P<0.001). The log_10_ VL at HIV diagnosis was significantly higher in non-controllers on HAART (4.8, IQR 4.2–5.1; P<0.001) and CD4 at both HIV diagnosis and last vaccination was lower (363, IQR 230–543 cells/uL; P<0.001 and 524, IQR 380–714 cells/uL; P = 0.023, respectively). Non-controllers on HAART also had a significantly higher proportion of AIDS events prior to last vaccination compared to HIC (38.7% vs 13.6%; P = 0.002). The time from HIV diagnosis to receipt of last HBV vaccination was significantly shorter for HIC (17 months, IQR 7–48) compared to non-controllers on HAART (42 months, IQR 17–107; P<0.001), but the proportion of participants who received ≥3 vaccine doses was similar (52.3% vs 45.8%; P = 0.521).

The median time from last HBV vaccination to anti-HBs measurement was 5.6 months (IQR 3.8–7.1) for HIC and similar to both HAART-naïve and treated non-controller groups (5.3 months, IQR 2.0–8.0; P = 0.782 and 5.5 months, IQR 3.1–8.3; P = 0.493, respectively). HIC had a greater proportion of HBV vaccine responders compared to HAART-naïve non-controllers (65.9% vs 36.6%; P<0.001); however, anti-HBs response was no different compared to non-controllers on HAART (59.9%; P = 0.549). To investigate whether these findings were associated with differences in VL set point, we also observed greater responsiveness in HIC compared to HAART naïve non-controllers with VL<10,000 copies/mL at last vaccination (29 of 44, 65.9% vs 26 of 85, 30.6%, respectively; P = 0.009). However, the sample size was not large enough to perform logistic regression analyses and this observed gap in vaccine responsiveness may be partly explained by higher median CD4 cell counts at last vaccination in HIC compared to non-controllers (617 cells/uL, IQR 520–761 vs 456 cells/uL, IQR 355–602; P = 0.006). In a subgroup analysis of participants with an anti-HBs assessment after a single dose of HBV vaccine, a larger proportion of HIC responded (13 of 17, 76.5%) compared to both non-controllers who were HAART-naïve (67 of 228, 29.4%; P<0.001) and those on HAART (40 of 117, 34.2%; P = 0.002).

Logistic regression analyses were performed to identify the factors associated with HBV vaccine response after last vaccination (Table 2). For HIC and HAART-naïve non-controller groups, HIC status was significantly associated with vaccine response (OR 2.65, 95% CI 1.23–5.89; P = 0.014) as was CD4 count at last vaccination (OR 1.28, 95% CI 1.15–1.45 for every 100 cells/uL; P<0.001). Receipt of ≥3 vaccine doses was inversely associated with vaccine response (OR 0.56, 95% CI 0.35–0.88; P = 0.011). AIDS diagnosis prior to last vaccination (OR 0.84, 95% CI 0.45–1.52; P = 0.567) was not associated with vaccine response nor were demographic characteristics. For comparisons between HIC and non-controllers on HAART, only CD4 count at last vaccination (OR 1.23 for every 100 cells/uL, 95% CI 1.1–1.38; P<0.001) was associated with HBV vaccine response and HIC status was no longer significant (OR 1.16, 95% CI 0.53–2.6; P = 0.718).

**Table 2:** Factors Associated with Positive HBV Vaccine Seroresponse for HIV Controllers Compared to Non-controllers With or Without Viral Load-Suppressive HAART.

Characteristic	HIV Controllers vsHAART-naïve Non-Controllers (OR, 95% CI)	P-value	HIV Controllers vs Non-Controllers on HAART(OR, 95% CI)	P-value
HIV Controller vs Non-Controller	2.65 (1.23–5.89)	0.014	1.16 (0.53–2.6)	0.718
African-American vs Caucasian	0.89 (0.54–1.47)	0.662	1.38 (0.79–2.41)	0.256
Hispanic/Other vs Caucasian	1.76 (0.91–3.4)	0.091	0.93 (0.43–2.02)	0.847
Female vs Male	1.44 (0.75–2.75)	0.269	1.14 (0.56–2.38)	0.717
Age at Last Vaccination(Per 10 years)	0.75 (0.54–1.04)	0.087	0.98 (0.76–1.26)	0.884
CD4 Count at Last Vaccination(For Every 100 Cells/uL)	1.28 (1.15–1.45)	<0.001	1.23 (1.1–1.38)	<0.001
Number of Vaccine Doses,≥3 vs 1–2	0.56 (0.35–0.88)	0.011	1.11 (0.66–1.86)	0.693
AIDS Before Last Vaccination	0.84 (0.45–1.52)	0.567	1.63 (0.91–3.0)	0.107

HBV, hepatitis B virus; HAART, highly active antiretroviral therapy; OR, odds ratio; CI, confidence interval; AIDS, acquired immunodeficiency syndrome.

Data are number (%) or median (Interquartile Range).

Positive HBV vaccine seroresponse defined as antibody to HBV surface antigen ≥10 IU/L measured ≥1 month after last HBV vaccination.

### Development of AIDS or Death by HBV Vaccine Response Status

A total of 143 HIC, comprised of 17 elite and 126 viremic controllers, and 1566 non-controllers met criteria for analysis (Table 3). The proportion receiving all HBV vaccines prior to HIV diagnosis for HIC and non-controllers was 22.4% and 23.2% (P = 0.895), respectively, with the remaining participants receiving 1 or more vaccine doses after HIV diagnosis. The median time from the start of HBV vaccination to HIV diagnosis was 42.5 months (IQR 18.6–82.3) for participants vaccinated prior to HIV diagnosis. Demographic characteristics were similar between HIC and non-controller groups. Compared to non-controllers, HIC had higher CD4 count (672 cells/uL, IQR 535–884 vs 460 cells/uL, IQR 342–624; P<0.001) and lower log_10_ VL (2.9, IQR 2.4–3.4 vs 3.8, IQR 2.6–4.5; P<0.001) at the time of HBV response categorization. Non-controllers had a higher proportion of prior AIDS events compared to HIC (17% vs. 5%, respectively; P<0.001). A positive HBV vaccine response occurred in 975 of 1566 (62.3%) non-controllers and 113 of 143 (79.0%) HIC, including 14 of 17 (82.4%) elite and 99 of 126 (78.6%) viremic controllers. Although the median follow-up was longer for HIC (7.4 years, IQR 4.2–14.0) compared to non-controllers (6.2 years, IQR 3.1–11.0; P = 0.055), HAART use was significantly less (42.7% vs 61.0%; P<0.001). There were no deaths and only 1 AIDS diagnosis in the HIC group, specifically a viremic controller with recurrent pneumonia categorized as a vaccine responder. In contrast, the composite outcome of AIDS events or death occurred in 165 of 1566 (10.5%) non-controllers, with a greater proportion of events observed in non-responders (110 of 591, 18.6%) compared to responders (55 of 975, 5.6%).

**Table 3:** Baseline Characteristics for HIV Controllers and Non-controllers for Time to AIDS or Death Analysis.

Characteristic	Total	HIV Controllers	Non-Controllers	P-value
Number of Participants (N)	1709	143	1566	
Age at HIV Diagnosis (Years)	28 (24–33)	28 (23–34)	28 (24–33)	0.558
Gender				0.243
Male	1504 (88.0%)	121 (84.6%)	1383 (88.3%)	
Female	205 (12.0%)	22 (15.4%)	183 (11.7%)	
Race/Ethnicity				0.413
European American	750 (43.9%)	56 (39.2%)	694 (44.3%)	
African American	714 (41.8%)	67 (46.9%)	647 (41.3%)	
Hispanic/Other	245 (14.3%)	20 (14.0%)	225 (14.4%)	
CD4 Count at HIV Diagnosis (Cells/uL)	494 (361–646)	654 (506–814)	480 (346–630)	<0.001
VL at HIV Diagnosis (Log_10_ Copies/mL)	4.4 (3.7–4.9)	3.2 (2.4–3.7)	4.5 (3.9–4.9)	<0.001
All HBV Vaccine Doses Before HIV Diagnosis	396 (23.2%)	32 (22.4%)	364 (23.2%)	0.895
CD4 Count at Vaccine Response Categorization (Cells/uL)	475 (350–646)	672 (535–884)	460 (342–624)	<0.001
VL at Vaccine Response Categorization (Log_10_ Copies/mL)	3.6 (2.6–4.4)	2.9 (2.4–3.4)	3.8 (2.6–4.5)	<0.001
AIDS Prior to Vaccine Response Categorization	280 (16.4%)	7 (4.9%)	273 (17.4%)	<0.001
Length of Follow-up (Years)	6.3 (3.2–11.0)	7.4 (4.2–14.0)	6.2 (3.1–11.0)	0.055
HAART Use During Follow-up	1016 (59.4%)	61 (42.7%)	955 (61.0%)	<0.001

VL, viral load; HBV, hepatitis B virus; AIDS, acquired immunodeficiency syndrome; HAART, highly active antiretroviral therapy.

Values are number (%) or median (Interquartile Range).

Positive HBV vaccine seroresponse defined as antibody to HBV surface antigen (anti-HBs) ≥10 IU/L following last HBV vaccination. For those vaccinated before HIV, the first available anti-HBs determination after HIV infection was used for categorization.

**Figure 1:**
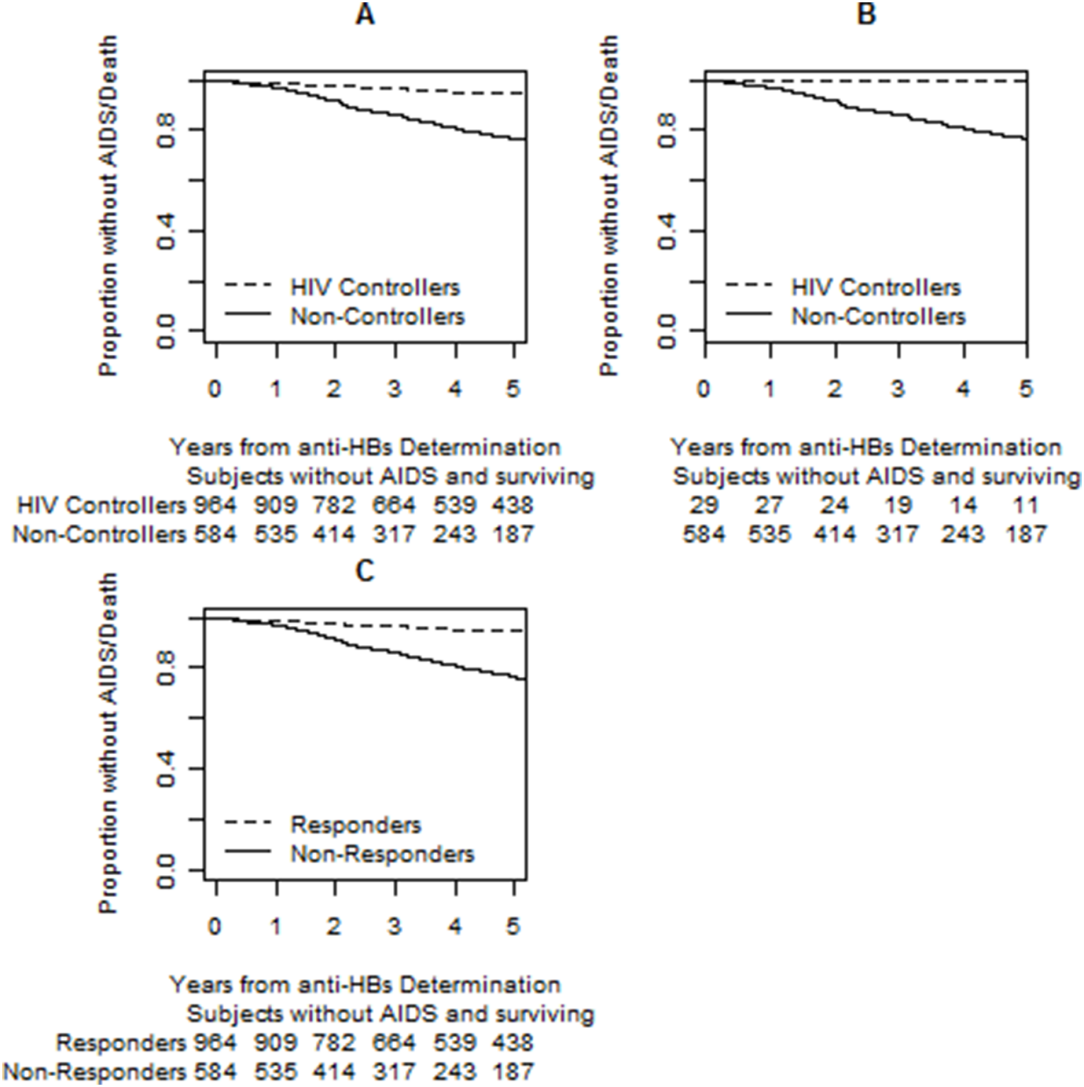
Kaplan-Meier plots demonstrating the time to AIDS or death for HIV controllers (HIC) compared to non-controllers by hepatitis B virus (HBV) vaccine antibody response status (A and B) and outcomes for non-controllers alone (C). Among HBV vaccine responders (A) and non-responders (B), the time to AIDS or death was significantly longer for HIC compared to non-controllers (Log Rank P<0.001 for both). (C). Examination of non-controllers alone by HBV response status showed a longer time to AIDS or death for vaccine responders compared to non-responders (P<0.001). Anti-HBs, antibody to HBV surface antigen.

The rate of AIDS or death events among HBV vaccine responders was 0.98 (95% CI 0.74–1.28) per 100 person/years for non-controllers compared to 0.14 (95% CI 0–0.76) per 100 person/years for HIC. For HBV vaccine non-responders, the rate for non-controllers and HIC was 4.11 (95% CI 3.38–4.96) and 0 (95% CI 0–2.22) per 100 person/years, respectively. Cox proportional hazards model showed a decreased risk of AIDS or death for HBV vaccine responders (HR 0.33, 95% CI 0.22–0.52) but not for HIC (HR 0.2, 95% CI 0.03–1.45) (Table 4). The risk of AIDS or death was also reduced for participants with higher CD4 counts at last vaccination (HR 0.8, 95% CI 0.69–0.93 for every 100 cells/uL). Kaplan-Meier plots demonstrate that the time to AIDS or death was significantly longer for HIC versus non-controllers regardless of whether participants were categorized as vaccine responders or non-responders (P<0.001 for both, Figure 1A and 1B). HBV vaccine response status stratified non-controllers for the outcome of AIDS or death as responders had a significantly longer time to this composite outcome compared to non-responders (P<0.001, Figure 1C). There were insufficient events to stratify HIC by HBV vaccine response status.

**Table 4:** Cox proportional hazard model for factors associated with risk of AIDS or death event.

Characteristic	Adjusted HR (95% CI)	P-value
HBV Vaccine Responder vs. Non-responder	0.33 (0.22–0.52)	<0.001
HIV Controller vs Non-Controller	0.2 (0.03–1.45)	0.111
African-American vs Caucasian	1.04 (0.70–1.54)	0.845
Hispanic/Other vs Caucasian	0.88 (0.49–1.57)	0.662
Female vs Male	0.56 (0.30–1.04)	0.065
Age at Last Vaccination (Per 10 years)	1.04 (0.81–1.34)	0.759
Number of Vaccine Doses, ≥3 vs 1–2	0.88 (0.61–1.28)	0.498
CD4 Count at Last Vaccination (For Every 100 Cells/uL)	0.80 (0.69–0.93)	0.003
Nadir CD4 Count Prior to Last Vaccination (For every 100 Cells/uL)	1.12 (0.94–1.34)	0.213
AIDS Before Last Vaccination	1.43 (0.9–2.29)	0.134

HR, hazard ratio; CI, confidence interval; HBV, hepatitis B virus; AIDS, acquired immunodeficiency syndrome.

Model stratified by era of HIV diagnosis (before or after 1996).

## Discussion

The analyses comparing non-controllers to HIC, a subgroup of HIV-infected individuals with natural suppression of plasma VL, showed that HIC had improved HBV vaccine seroresponses compared to treatment-naïve non-controllers, but the deficiency in vaccine responsiveness observed in non-controllers was mitigated by use of VL-suppressive HAART. This finding is in agreement with prior studies demonstrating that HAART improves HBV vaccine responses in HIV-infected persons [13], [16], [17]. HIC were also more likely to respond to a single dose of HBV vaccine compared to non-controllers regardless of HAART use. These results suggest that HIC may have certain immunologic characteristics that lead to enhanced HBV vaccine responsiveness. Despite these favorable characteristics, HIC have diminished responsiveness compared to HIV-uninfected persons (66% vs. 90%). The clinical importance and prognostic value of HBV vaccine response was also reconfirmed in this study with vaccine responders having a lower proportion and risk of AIDS events or death, though this was mainly evident in non-controllers as outcomes were exceedingly rare in HIC.

In a previous study in our cohort, a greater likelihood of HBV vaccine response was demonstrated in those with higher CD4 cell counts at vaccination and with use of HAART [13]. In our current study, higher CD4 count at last vaccination was associated with vaccine response for analyses with non-controllers with or without use of HAART. However, the combination of robust immune reconstitution on HAART with suppressed VL appears to have a considerable impact on HBV vaccine responses in several studies [13], [21], [22]. For example, in a previous study in our cohort, 62% of participants with CD4>350 cells/uL and VL<400 copies/mL on HAART were responders; a stark contrast to the 20% response observed for those with CD4<350 cells/uL in the absence of HAART [13].

Interestingly, in this study we found that among participants with anti-HBs determinations available after the first dose of HBV vaccine, HIC had greater responses (76%) compared to non-controllers with and without HAART (34%; P = .002 and 29%; P = .001, respectively). HIC may have a more rapid response to HBV vaccination compared to non-controllers, however other factors may have contributed to this observation such as CD4 at first vaccination. For those completing the vaccine series, our previous study showed that receipt of ≥3 vaccine doses was associated with a positive response [13]. This finding was attributed to HAART increasing the likelihood of response for those characterized as non-responders prior to HAART initiation. Our current study showed an inverse association between number of vaccine doses and anti-HBs response only in HAART-naïve non-controllers as additional doses did not improve responsiveness in the absence of HAART.

To further emphasize the importance of HAART for HIV-infected individuals, we observed greater HBV vaccine responsiveness in HIC compared to HAART-naïve non-controllers but similar to those on HAART in our current study. HBV vaccine responsiveness in HIV-infected persons has been associated with suppressed VL, however this has only been reported in the setting of HAART and not in those with natural suppression of HIV viremia. For example, Overton et al [16] reported plasma VL<400 copies/mL as the only factor associated with a protective anti-HBs response among a group of 194 HIV-infected adults. For children with prior non-response to HBV vaccine, initiation of VL-suppressive HAART was associated with successful anti-HBs response upon re-vaccination [23]. A study by Fonseca et al [24] reported that vaccine response was significantly improved when VL<10,000 copies/mL, however half of the study population was on HAART. When compared to HAART naïve non-controllers with VL<10,000 copies/mL at last vaccination in our current study, a higher proportion of HIC were vaccine responders (65.9% vs 30.5%, respectively; P = 0.009). Based on our findings and those of previous studies, VL suppression by treatment with HAART or natural suppression in HIC is associated with greater HBV vaccine responsiveness.

Since HIC status is associated with favorable immunologic characteristics, enhanced functional immunity may play a role in the improved HBV vaccine seroresponses observed in this group. There are numerous processes and pathways involved in the development of antibodies, some of which differ in HIC compared to non-controllers. For example, interleukin-4 (IL-4) and IL-10 are important regulators of antibody response [25], [26]. Basal IL-10 levels are reduced in HIV-infected persons and those with advanced disease have both delayed and diminished IL-10 responses following HBV vaccination [27]. A recent study showed that elite and viremic controllers had relatively preserved IL-10 production compared to typical progressors and viremic long term non-progressors which may contribute in part to the enhanced HBV vaccine seroresponses observed in HIC [28].

HIV infection in the absence of HAART has been shown to result in B-cell defects including increased expression of activation markers, higher risk of B-cell lymphomas, increased autoantibody levels, and decreased responsiveness to vaccination [29]. The absence of HBV vaccine response may be secondary to the inability of CD4+ T-cells to activate B-cells into isotype switching of immunoglobulins due to diminished expression of CD40L in persons with HIV [9]. CD40 ligand (CD40L) signaling is critical for many immunologic functions, including antibody production, surface molecule upregulation, isotype switching, germinal center development, and humoral memory response [30], [31]. A study by Whittall et al [32] demonstrated that the addition of CD40L and IL-4 *in vitro* were most effective in converting the immunologic signatures of non-controllers to those observed in HIC. Regulatory T-cells (T_regs_) also play an important role in modulating immune responses and HBV vaccine response was found to be associated with lower baseline T_reg_ frequency in HIV-infected persons [33]. Individuals with VL suppression, either naturally like HIC or by HAART, have lower levels of T_regs_ compared with untreated persons with HIV [34]. The observation that VL-suppressive HAART effectively closes the HBV vaccine response gap between non-controllers and HIC may be explained in part by the amelioration of these T- and B-cell defects during effective HAART.

The prognostic value of positive HBV vaccine seroresponses was also reconfirmed in our study with the observation of both decreased risk and proportion of AIDS and death events during long-term follow-up for both HIC and non-controllers. In a previous study in our cohort, HBV vaccine non-responders had a 4-fold higher rate of developing AIDS or death compared to non-responders [13]. The independent association between HBV vaccine responses and AIDS or death was even observed in the subset participants with CD4 count >500 cells/uL at last vaccination. Since this association was observed in those with relatively preserved CD4 counts, we investigated whether HBV vaccine responsiveness could be used as a predictor for HIV disease progression in HIC. Similar to our previous study in HIC, the approximate median CD4 cell count at HIV diagnosis was 650 cells/uL [1]. Although HIC had a significantly longer time to development of AIDS or death compared to non-controllers, there were insufficient AIDS or death outcomes to stratify HIC by HBV vaccine response. However, a positive HBV vaccine response among non-controllers predicted slower HIV disease progression and a 67% reduction in the risk of AIDS or death compared to vaccine non-responders.

Limitations of this study include the small number of AIDS and death events, particularly for HIC. As a retrospective cohort study, the timing and administration of HBV vaccine and HAART were not randomized and the dose of HBV vaccine administered was unavailable. Although the sample size of the HIC group was appropriate for statistical comparisons of vaccine responses, participants were predominantly viremic controllers and we were unable to directly compare elite and viremic controller subgroups.

In summary, a positive response to HBV vaccine is important for HIV-infected individuals for prevention of liver-related morbidity due to HBV infection and may also have prognostic value for predicting HIV disease progression. HIC status is associated with greater overall HBV vaccine responsiveness compared to untreated non-controllers, but similar to those on VL-suppressive HAART. HIC are more likely to respond to a single dose of HBV vaccine compared to non-controllers regardless of HAART use, but the clinical significance of rapid vaccine response to HBV vaccine has not been formally studied. Additional *in vivo* and *in vitro* studies of vaccine response are warranted to further understand functional immunity in HIC.
